# A cross‐sectional survey to explore the prevalence and causes of occupational burnout syndrome among perioperative nurses in Saudi Arabia

**DOI:** 10.1002/nop2.637

**Published:** 2020-10-04

**Authors:** Bader Ali Almodibeg, Hazel Smith

**Affiliations:** ^1^ School of Healthcare Sciences Operating Department Practice Cardiff University Cardiff UK

**Keywords:** occupational burnout syndrome, operating room, perioperative nurses, Saudi Arabia

## Abstract

**Aim:**

To detect the level of burnout and its most significant causes among perioperative nurses.

**Design:**

A descriptive quantitative cross‐sectional survey design.

**Methods:**

Data on burnout and its most significant causes were collected by surveying 39 perioperative nurses in a regional hospital in Saudi Arabia using the Maslach Burnout Inventory and a self‐developed questionnaire. Descriptive statistics were used to perform statistical analysis.

**Results:**

Burnout was detected in 5% of respondents. A high level of emotional exhaustion was detected in 87.2%. Similarly, a high level of depersonalization was detected in 56.4%, while 15.4% of nurses showed a low sense of personal accomplishment. Several factors were identified as the causes of burnout such as high workload, staff shortage, poor teamwork, insufficient salary and occupational hazards. However, lack of departmental support and undesirable supervision in the workplace seem to be the main causes of burnout.

## 1 INTRODUCTION

It is projected that the world will face a shortage of healthcare practitioners of 7.2 million by 2035 (WHO, 2018). The World Health Organization (WHO) warns that this shortfall will have serious implications for the health of billions of people around the world if not addressed. Saudi Arabia is one country that is suffering from this shortage of healthcare practitioners, especially nurses, as it mainly relies on recruiting and employing international nurses, who represent 64% of the nursing workforce (Ministry of Health, 2017).

The International Council of Nurses (ICN) suggests that there are several causes contributing to the present shortage of nurses around the world (ICN, 2006). The main cause is the high rate of nurses leaving the profession due to poor working conditions, job dissatisfaction and what has been termed Occupational Burnout Syndrome (OBS). Therefore, the awareness and causes of OBS is extremely important to implement effective workforce strategies that promote practitioner well‐being, reduce absenteeism, increase practitioner satisfaction and decrease turnover rate (Espeland, 2006).

The classic definition of Occupational Burnout Syndrome is “a psychological syndrome of emotional exhaustion, depersonalization or cynicism and inefficacy, which is experienced in response to chronic job stressors” (Maslach et al., 2001, p. 333).

Emotional exhaustion (EE) refers to “feelings of physical, emotional and mental exhaustion from one’s work” (Maslach et al., 2016, p. 6). Workers experiencing emotional exhaustion (EE) distance themselves from their task, both emotionally and cognitively, in an attempt to cope with their work stressors (Schaufeli et al., 2009). This leads to “depersonalization” (DP) whereby workers develop apathy or cynical behaviour (Schaufeli et al., 2009). The third component of OBS is the lack of personal accomplishment (PA), which refers to a sense of inefficiency, unproductivity and lack of successful achievement in the workplace (Schaufeli et al., 2009).

Maslach et al. (2001), who have undertaken significant research in this phenomenon, characterized OBS as having three component dimensions, namely high levels of EE and DP, along with a lack of PA.

Schaufeli et al., 2009 reviewed the research on OBS over the past two decades and considered the following areas of work life as critical sources of OBS:

- Overload
- Lack of independence in planning and organizing tasks
- Insufficient rewards and resources in workplace
- Unfairness in practice
- Poor teamwork
- Lack of support.

## 2 BACKGROUND

There is a plethora of international literature identifying the prevalence rates and causes of OBS among nurses in different departments (Adriaenssens et al., 2015; Khamisa et al., 2013; Parola et al., 2017). However, a comprehensive literature search found only a limited number of studies exploring the prevalence rate and causes of OBS among perioperative nurses.

In Spain, Sillero and Zabalegui (2018) explored the prevalence rate of OBS among 136 perioperative nurses and found that 41% of those nurses had OBS. However, unfortunately, the authors of this study exaggerated the prevalence rate of OBS by adding participants who showed only high levels of EE and DP as well as participants who had full‐blown OBS (high levels of EE and DP along with a low level of PA). Another study, conducted by Khorasani Niasar et al. (2013), aimed to detect the levels of the three OBS dimensions among 87 perioperative nurses working in one of the biggest tertiary hospitals in Iran. This study showed that 45% of those nurses had high levels of EE, while 16% had high DP. Interestingly, all (100%) of the participants had low levels of PA. Khorasani Niasar et al. (2013) determined that there was a statistically significant relationship between the female gender and high levels of EE and DP (*p* = .03, *p* = .04, respectively). In addition, there was a significant correlation between workload and higher DP (*p* = .05).

In Turkey, Findik’s (2015) multicentre cross‐sectional survey, with an acceptable response rate of 60% (*n* = 64), found that 100% of perioperative nurses had low levels of PA, while 32% had high scores in both EE and DP. Findik (2015) found that EE and DP increased in perioperative nurses who felt that they were unsupported by their senior management (*p* = .002, *p* = .009, respectively). Interestingly, Findik (2015) investigated more specific perioperative issues and found that EE increased in perioperative nurses who failed to take necessary precautions to prevent sharp injuries (*p* = .005), while DP increased in those who failed to take necessary precautions in areas where radiation was used (*p* = .008).

In the context of Saudi Arabia, the Ministry of Health is working hard to improve the effectiveness of health workforce strategies to maintain practitioners’ well‐being and decrease the rate at which they leave the profession (Elsheikh et al., 2018). However, it could be difficult to predict how effective these workforce strategies will be, since awareness of OBS and research into the prevalence and causes of OBS in nursing are relatively low in Saudi Arabia (Haifa, 2010). This is reflected in the fact that the literature search only resulted in two studies being found.

Haifa (2010) used the validated survey compiled by Maslach in 2006, known as the Maslach Burnout Inventory, to collect their data. Thirty‐seven female Saudi nurses working in a tertiary centre responded, and although this was a small study, it indicated that 70% of the nursing staff suffered from OBS. However, caution must be shown in accepting these results, as the high prevalence rate can be explained due to the author only considering the presence of one high level score in EE or DP or one low score in PA as evidence to diagnose OBS. As Maslach et al. (2016) stated, OBS can only be diagnosed when all three dimensions are used and when there are high levels of EE and DP along with low levels of PA.

Al‐Turki et al. (2010) aimed to detect the levels of all three OBS dimensions among 198 multinational nurses working in the same centre in Saudi Arabia. The study showed that 46% of participating nurses had high levels of EE, 42% had high levels of DP and 28% had low levels of PA. Al‐Turki et al. (2010) reported that there was significantly higher EE and DP among nurses working in the wards compared with nurses working in critical areas, such as operating rooms.

As neither OBS prevalence among perioperative nurses nor contributing factors are known in the context of Saudi Arabia, this exploratory study will give insights into burnout and identify its causes, thus helping to formulate evidence‐based strategies to take appropriate action to manage burnout by tackling the causative factors.

### 2.1 Research question

What are the prevalence rate of OBS and the most significant causes of OBS among perioperative nurses staff working in the OR of a regional hospital in Saudi Arabia?

## 3 METHODS

### 3.1 Design

A descriptive cross‐sectional questionnaire survey.

### 3.2 Study sample and setting

In this study, purposive sampling was used to recruit all perioperative nurses who worked full time in the OR of a regional hospital in Saudi Arabia. Students and part time workers were excluded. This centre was selected because it is a regional, trauma and referral hospital covering one province of Saudi Arabia. This hospital employs 42 perioperative nurses, of whom 28 (69%) are international nurses.

### 3.3 Data collection

Envelopes containing self‐reported questionnaire were distributed by researchers to all participants who given three weeks to complete the questionnaires and return them in a sealed envelope to the collection area.

### 3.4 Questionnaire

The questionnaire consisted of three sections of questions. The first section gathered demographic information. The second section included the Maslach Burnout Inventory (MBI) to detect the prevalence of OBS. The MBI questionnaire contains 22 closed‐ended rating items which are presented in the format of statements to capture personal feelings and attitudes. The respondent is asked to indicate how often each statement occurs on a scale ranging from 0 (never) to 6 (every day) (Maslach et al., 2016). Nine of the 22 items in this questionnaire measure the respondent’s EE, while five items measure DP and eight items measure PA. The scores for these dimensions are determined using the following equation: how often [0 to 6] × the number of dimension items (Maslach et al., 2016). OBS is diagnosed when the respondent gets high scores for EE and DP and a low score for PA (Maslach et al., 2016). To define the cut‐off scores indicating high, moderate and low levels in each dimension, Maslach et al. (2016) supported using nation‐specific cut‐off points to help researchers to compare and know more about the prevalence of OBS in these specific national groups. Therefore, the predetermined cut‐off scores that were used in the studies conducted in Saudi Arabia by Haifa (2010) and Al‐Turki et al. (2010) were used in this study to define the levels of OBS dimensions (See Table 1). The validity and reliability of the MBI have been confirmed among perioperative nurses and even in the context of Saudi Arabia (Al‐Turki et al., 2010; Khorasani Niasar et al., 2013). The reliability coefficients ranged from 0.85–0.89 for EE statements, from 0.83–0.84 for DP statements and from 0.86–0.90 for PA statements.

**TABLE 1:** Level of OBS dimensions

OBS Dimensions level	Cut‐off point*	Job title of participants	
		Nurse	
		Count	Column Total *N* (%)
EE level			
Low	˂18	2	5.1
Moderate	18–26	3	7.7
High	˃26	34	87.2
Total	39	100.0%	
Level of DP			
Low	˂7	5	12.8
Moderate	7–12	12	30.8
High	˃12	22	56.4
Total	39	100.0%	
Level of PA			
High	˃36	24	61.5
Moderate	31–36	9	23.1
Low	˂31	6	15.4
Total	39	100.0%	

* Cut‐off scores derived from normative data in Saudi Arabia.

The third section of this study’s questionnaire was designed by the researcher to explore the causes of OBS by seeking to identify the respondents’ perceptions of the causes of their OBS. This question includes the 13 main causes of OBS which the literature review showed to be contributing to OBS, particularly in nurses. These causes were stated in closed‐ended rating format, and the respondents were asked to rate the significance of each on a scale ranging from 1 (least significant) ‒ 3 (most significant) if they thought that these causes were contributing to their OBS. Nonetheless, to gain more detail about the respondents’ perceptions about the causes of OBS, the researcher partially opened this question by adding five spaces to give the respondents the opportunity to add other causes and rate their significance. The face validity of this questionnaire was confirmed by an expert researcher and by two perioperative nurses who have extensive experience in the OR environment. The reliability of this questionnaire was also confirmed by piloting it with two perioperative nurses who worked in the operating room of the hospital where this study was to take place. Those respondents and their responses were excluded from the main study’s sample.

### 3.5 Statistical analysis

Descriptive statistics via SPSS version 23 Software were used to perform statistical analysis.

Sociodemographic and occupational information data are considered as nominal data, and it was reported as frequencies. The MBI consists of interval scales (Maslach et al., 2016) and was thus treated as scale data and reported as means and standard deviations. The causes of OBS consist of an ordinal scale, which was accordingly treated as ordinal data and then the most significant causes were transformed and recorded into different variables to be reported as percentages and absolute numbers.

Descriptive statistics, including these procedures (frequencies, descriptive, crosstabs and explore), were used as appropriate. Participants with high scores in EE and DP alongside with low score in PA were transformed and recorded into different variables to be reported as percentages and absolute numbers.

### 3.6 Ethics

Research Ethics Committee approval for this study was gained from the Ethics and internal review board at the hospital where the study was conducted and from the school’s research ethics committee at Cardiff University. Prior to data collection, the researcher distributed a written consent form and an information sheet to each respondent explaining the aim, benefits and risks of the study, the purposed use of the data collected and information on anonymity and confidentiality.

Anonymity and confidentiality of respondents were ensured, as the researcher distributed envelopes where the respondents placed the survey, then sealed it and placed it in a sealed box at the front of a changing room. In addition, respondents’ names were not required in the study questionnaires.

### 3.7 Results

Forty questionnaires were administered to perioperative nurses after excluding the pilot study participants. Thirty‐nine of these questionnaires were completed and returned, giving a response rate of 97.5%. Most perioperative nurses were females (89.7%), and 69.2% of the nurse sample was non‐Saudi. Regarding work experience, the perioperative nurses’ sample comprised 35.8% junior nurses, while senior nurses represented 33.3% of the sample and 30.8% of perioperative nurses had middle experience.

OBS was detected in 5% of perioperative nurses, while the remaining 95% did not have full‐blown OBS. However, EE was high in most perioperative nurses (87.2%), while high levels of DP were detected in 56.4%. The PA level was high in almost two‐thirds of the perioperative nurses (61.5%), while a low level was found in only 15.4% of the perioperative nurses (Table 1).

**FIGURE 1:**
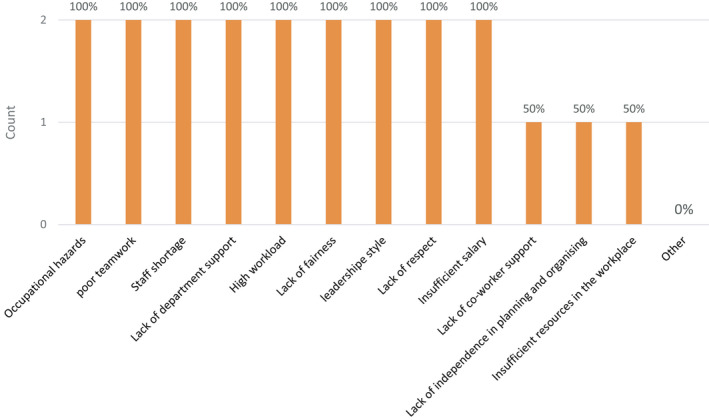
The most significant causes of OBS in perioperative nurses

Regarding the most significant causes of OBS, respondents were asked to rate the above main OBS causes in order of significance in causing their OBS, from least significant to most significant. All perioperative nurses who had OBS (100%, *N* = 2) perceived all of the above as “most significant” causes of their OBS except “Lack of co‐worker support,” “Lack of independence in planning and organizing work” and “Insufficient resources in the workplace,” which were only perceived to be “most significant” by 50% (*N* = 1) of perioperative nurses who had OBS. No other causes were specified (Figure 1).

## 4 DISCUSSION

### 4.1 Levels of OBS dimensions

Detection of the levels of the three OBS dimensions is necessary not only to identify the prevalence of OBS but also to detect the respondents’ various workplace experiences (Maslach et al., 2016). EE was detected at a high level in most perioperative nurses who participated in this study (87.2%). This result contradicts a study by Khorasani Niasar et al. (2013), who found that only 45% of Iranian perioperative nurses had high levels of EE. The differences in the sample composition between this study and that of Khorasani Niasar et al. (2013) might lead to this contradiction. In this study, 69.2% of respondents were foreign nurses. Al‐Turki et al. (2010) found that foreign nurses were significantly more prone to EE than local nurses in Saudi Arabia (*p* = .004). Homesickness and cultural differences could contribute to the risk of a higher level of EE (Al‐Turki et al., 2010).

DP is another dimension of OBS that results from protracted exhaustion. More than half of the perioperative nurses who participated in this study (56.4%) had high levels of DP. Morais et al. (2006) argued that due to the characteristics of the speciality, OR staff are not closely involved in relationships with patients, with little contact or follow‐up after the patients leave the OR, which can lead them to develop loss of empathy and subsequently depersonalization. This argument is supported by Haifa (2010), who found that 49% of multi‐department nurses working in Saudi Arabia had high levels of DP and that there was significantly higher DP among nurses working in critical care departments compared with nurses working in the wards (*p* = .003).

The third dimension of OBS is viewed as the lack of PA. Perioperative nurses in this study showed a high sense of PA, as only 15.4% had lack of PA. The low prevalence of lack of PA detected among perioperative nurses in this study is in the range detected among nurses in Saudi Arabia by Al‐Turki et al. (2010) and Haifa (2010). However, both Khorasani Niasar et al. (2013) and Findik (2015) found that 100% of non‐foreign perioperative nurses who participated in their studies reported a lack of PA. Arguably, the foreign perioperative nurses in this study feel that they are accomplishing many worthwhile things through working in Saudi Arabia. This argument is supported by the current study’s findings, as non‐Saudi nurses had higher PA than Saudi nurses.

### 4.2 Prevalence of OBS among perioperative nurses

The prevalence of OBS among perioperative nurses was low, being detected in only 5% of nurse respondents. However, as there is a lack of literature that reports full‐blown burnout syndrome among perioperative nurses, this result cannot be compared with prior findings. Nevertheless, the perioperative nurses in this study had a critically higher risk of OBS in comparison with the studies reviewed. Sillero and Zabalegui (2018) reported that 41% of perioperative nurses in Spain had moderate or high risk of OBS. In this study, most perioperative nurses (84.6%) had moderate or high risk of OBS. Similarly, in the context of Saudi Arabia, Haifa (2010) showed that 70% of Saudi nursing staff working at one centre in Saudi Arabia had at least one high score in EE or DP or one low score in PA, while this study showed that 97.4% of perioperative nurses had at least one high score in EE or DP or one low score in PA.

### 4.3 The most significant causes of OBS in perioperative nurses

In this study, the participants were asked to rate the significance of various causes of OBS identified from the literature. All perioperative nurses who had OBS rated “Occupational hazards” as one of the most significant causes of their OBS. The contribution of occupational hazards to OBS was also detected by Findik (2015), who found that EE increased in perioperative nurses who failed to take necessary precautions to prevent sharp injuries (*p* = .005), while DP increased in perioperative nurses who failed to take necessary precautions in areas where radiation is used (*p* = .008).

There was a strong perception among perioperative nurses who participated in this study that there was a lack of departmental support and undesirable supervision, which also contributed to their OBS. Arguably, the lack of departmental support could lead to insufficient resources and staffing in the workplace, which in turn could lead to OBS. In addition, staff shortage would not only contribute to OBS but also exacerbate the high workload of staff, which in turn could also lead to OBS. This argument is apparent from the current study results, as all perioperative nurses with OBS rated “Staff shortage” and “High workload” as the other most significant causes of their OBS, while insufficient resources in the workplace were perceived with lesser severity, as only half of perioperative nurses with OBS rated “Insufficient resources in the workplace” as one of the most significant causes of their OBS.

Another factor causing OBS that was detected by the current study is insufficient rewards, which could be a consequence of lack of departmental support. In this study, perioperative nurses who had OBS perceived “Insufficient salary” as one of the most significant causes of their OBS. As all perioperative nurses who had OBS were non‐Saudi, the wage gap between health workers in Saudi Arabia may contribute to OBS among expatriate practitioners. The ethnic wage gap among the health workforce is a global issue even in developed countries (NHS Workforce Statistics, 2018). The contribution of such wage gaps to OBS is totally unknown and requires further investigation. Arguably, the wage gap among healthcare workers would reinforce feelings of unfairness, which in turn could also lead to OBS. This argument is supported by the current study’s findings, which show that all perioperative nurses with OBS rated “Lack of fairness” as one of the most significant causes of their OBS. However, unfairness is not limited to the wage gap, as it can also include supervisors’ unfair practice in aspects such as duty and off‐duty distribution and on‐call duties.

Unfairness can also contribute to poor teamwork (Khalib & Ngan, 2006), which in turn can cause OBS. This is apparent from the current study’s results, as “Poor teamwork,” “Lack of co‐worker support” and “Lack of respect in the workplace” were rated by most perioperative nurse with OBS as significant causes of their OBS.

The last factor causing OBS that was detected by the current study is the lack of independence in planning and organizing work, which was rated by half of the perioperative nurses who had OBS as a “most significant” cause of their OBS. The healthcare system in Saudi Arabia relies heavily on physician‐based care, while non‐physician practitioners have a limited career pathway (Walston et al., 2008). Although this strategy has been adopted to enhance patient safety, the risk of OBS among non‐physician practitioners could rise, which threatens patient safety from the other side. Therefore, implementing physician‐led team‐based care where the tasks are redistributed among healthcare staff and involve non‐physician practitioners in patient care, such as taking health histories and undertaking pre‐assessment and postvisit care, would reduce the risk of burnout among non‐physician practitioners and would also free up the physicians to focus more on difficult tasks in patient care (Smith et al., 2018).

**FIGURE 2:**
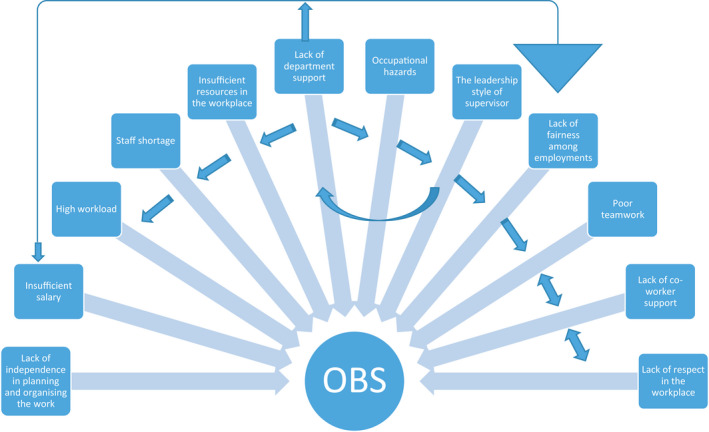
The relationships between the causes of OBS

Overall, this study results provide new evidence showing the prevalence and the most significant causes of OBS among OR nurses in Saudi Arabia. This evidence can be used in further qualitative research. In addition, some relationships were noted between the causes of OBS detect by this study, whereas each factor mentioned above makes a standalone contribution to OBS. We argue that the main predictors of the causes of OBS are the lack of departmental support and undesirable supervisor in the workplace. This is main theoretical contribution that may add to the literature. Lack of departmental support is likely to lead to insufficient resources and staffing shortages in the workplace. Staff shortages in turn would contribute to high workload. Also, the lack of departmental support or unfair supervisors could lead to unfair practice, whether in support, such as salaries, or in work demands, such as duties. Thus, lack of fairness in the workplace would lead to poor teamwork. Lastly, the lack of independence in planning and organizing the work could have some relationship to poor teamwork (Figure 2). However, the contribution of lack of departmental support and undesirable supervisor as the main predictors of the other causes of OBS need to be investigated by further studies. Such study would help to minimize the number of interventions and save time.

### 4.4 Study limitation

This study is limited, as it was conducted in a single selected hospital using a self‐administered method and recruited a relatively small sample: thus, there could be a risk of selection bias and response bias and generalizability to all perioperative nurses working elsewhere in Saudi Arabia cannot be confirmed. In addition, the current study used a quantitative approach to detect the causes of OBS. Thus, this study should be repeated by conducting a multicentre study using a mixed methods approach to detect the prevalence and causes of OBS among perioperative nurses in Saudi Arabia. Such study would generate results that could be generalizable to all perioperative nurses in Saudi Arabia.

## 5 CONCLUSION

Awareness of the prevalence and causes of OBS among perioperative nurses is extremely significant to improve effective workforce strategies that maintain practitioners’ well‐being. Our results show that the prevalence of OBS was low in perioperative nurses working in one centre in Saudi Arabia. Nevertheless, almost all of them are at high risk of OBS due to the feeling of exhaustion. Several factors were found to be highly significant causes of OBS. However, the main predictors of the causes of OBS among perioperative nurses seem to be the lack of departmental support and undesirable supervision in the workplace.

### 5.1 Recommendations for practice

- Payment of hazard pay to all perioperative nurses is recommended, as it would play a significant role in decreasing the risk of OBS‐related to occupational hazards.
- Sources of occupational hazards should be identified and the relevant department should implement safety training to minimize the risk of OBS among perioperative nurses.
- It is recommended that supervisors of perioperative nurses be well trained to be skilled, supportive and fair supervisors.
- Departments and managers must ensure that sufficient resources and staffing are in place in the workplace, as these would play a significant role in decreasing excessive workload and the risk of OBS among perioperative nurses.
- Equity in pay regardless of nationality would enhance the feeling of fairness, sufficient reward and teamwork, all of which in turn can minimize the risk of OBS.
- It is recommended that the decision‐making powers of perioperative nurses be increased by implementing physician‐led team‐based care so that they can be involved in patient care, as this would enhance the feeling of effectiveness, thus minimizing the risk of OBS.

## ETHICS

Research Ethics Committee approval for this study was gained from the Ethics and internal review board at the hospital where the study was conducted and from the schools research ethics committee at Cardiff University.

## CONFLICT OF INTEREST

The authors declare that there is no conflict of interest.

## Data Availability

The data used to support the findings of this study are available from the corresponding author on reasonable request.

## APPENDIX 1

### The literature search journey

**Table:**

Keywords		Database searched & results		
		Medline via Ovid	CINAHL	Scopus
Frist search keywords	“operating room” OR “operating theatre” OR “operating department” OR Perioperative AND Nurse* OR Technician* OR Technologist* OR practitioner OR “ODP” AND “Burnout syndrome” OR Burnout OR “Occupational Burnout syndrome” OR “Occupational Burnout” AND Causes OR factors OR “Contributing factors” OR “Influence factors”	11	7	16
Second search keywords	Nurse* OR Technician* OR Technologist* AND “Burnout syndrome” OR Burnout OR “Occupational Burnout syndrome” OR “Occupational Burnout” AND Saudi Arabia	5	3	5
